# Relationship between severe radiodermatitis and skin barrier functions in patients with head and neck cancer: A prospective observational study

**DOI:** 10.1016/j.apjon.2024.100625

**Published:** 2024-11-19

**Authors:** Nao Miyamae, Kazuhiro Ogai, Mao Kunimitsu, Masayuki Fujiwara, Makoto Nagai, Shigefumi Okamoto, Mayumi Okuwa, Makoto Oe

**Affiliations:** a Graduate School of Medical Sciences, Kanazawa University, Kanazawa, Japan; b Department of Fundamental Nursing, School of Nursing, Hyogo Medical University, Kobe, Japan; c Department of Bio-engineering Nursing, Graduate School of Nursing, Ishikawa Prefectural Nursing University, Kahoku, Japan; d Institute of Medical, Pharmaceutical and Health Sciences, Kanazawa University, Kanazawa, Japan; e Department of Radiology, School of Medicine, Hyogo Medical University, Nishinomiya, Japan; f Department of Dermatology, School of Medicine, Hyogo Medical University, Nishinomiya, Japan; g Department of Clinical Laboratory and Biomedical Sciences, Division of Health Sciences, Graduate School of Medicine, Osaka University, Suita, Japan

**Keywords:** Radiodermatitis, Head and neck cancer neoplasms, Skin care, Skin barrier function

## Abstract

**Objective:**

Severe radiodermatitis with erosion is a painful condition that affects quality of life; therefore, developing methods for its prevention is an urgent issue. Therefore, this study aimed to determine the morphological characteristics of the development and healing processes of severe radiodermatitis in patients with head and neck cancer and to explore the association between skin barrier function and development of severe radiodermatitis.

**Methods:**

In this prospective observational study, the cervical regions of patients with head and neck cancer who underwent radiotherapy at a university hospital from October 2022 to March 2023 were photographed, and morphological characteristics of the development and healing process of severe radiodermatitis were extracted using the qualitative sketch method. Skin barrier function, including skin microbiota and dermal echogenicity, was investigated before initiating radiotherapy, and its relationship with radiodermatitis was examined using the Mann–Whitney *U* test or Fisher's exact probability test.

**Results:**

Nine patients were followed for a median of 61 (range 55–87) days with a total of 88 observations. The morphological characteristics of severe radiodermatitis were “localized erosion–epithelialization” and “widespread erosion–crusting,” and compared to non-severe radiodermatitis, with low levels of *Staphylococcus aureus* (*P* ​= ​0.024), *Staphylococcus hominis* (*P* ​= ​0.024), and reduced dermal echogenicity (*P* ​= ​0.036). Furthermore, the “widespread erosion–crusting” was associated with a subepidermal low echogenic band.

**Conclusions:**

To prevent severe radiodermatitis, in addition to moisturizing the irradiated area and protecting it from mechanical irritation, improving skin barrier function before radiotherapy initiation may be effective.

## Introduction

Head and neck cancer encompasses cancers of the oral cavity, larynx, nasopharynx, oropharynx, and hypopharynx, with approximately 880,000 cases reported worldwide in 2020.^1^ Radiotherapy is generally indicated as an appropriate treatment method for head and neck cancer. Patients with head and neck cancer undergoing radiotherapy are prone to develop radiodermatitis, with about 25% developing severe radiodermatitis,^2^^,^^3^ which is painful owing to erosions and affects the patient's quality of life.^4^ Therefore, these patients require significant nursing care.

The risk factors influencing severe radiodermatitis are radiotherapy or patient-related. The radiotherapy factors are total dose, surface dose, irradiation technique, and use of radiosensitizers.^5^^,^^6^ Radiodermatitis severity depends on the total dose, with symptoms worsening when doses exceed 40 Gy, leading to desquamation and erosion due to skin stem cell death. Radiotherapy for head and neck cancer often involves doses > 60 Gy, which result in high surface irradiation due to its proximity to the skin, thus increasing the risk of severe radiodermatitis. Despite the use of novel techniques like Intensity Modulated Radiotherapy and Volumetric Modulated Arc Therapy (VMAT), severe radiodermatitis still occurs. Additionally, the concurrent use of chemotherapy or targeted agents in head and neck cancer radiotherapy can exacerbate radiodermatitis by inhibiting DNA repair and delayed epidermal cell regeneration.^7^ Patient-related factors such as age, sex, body mass index (BMI), cancer stage, smoking history, sun exposure, nutritional status, diabetes, and autoimmune diseases also contribute to radiodermatitis severity.^2^^,^^3^^,^^8^ Older patients with head and neck cancer,^7^ cancer stage IV,^7^ smoking,^2^ and poor nutritional status^8^ are more likely to develop severe radiodermatitis.

Skin barrier functional impairment at the irradiated site can lead to severe radiodermatitis. Patients with risk factors have more severely impaired skin barrier function and radiodermatitis. Radiation therapy-mediated changes in epidermal function include decreased sebum^9^ and stratum corneum moisture levels,^10^ increased transepidermal water loss (TEWL),^10^ decreased diversity of skin microbiota,^11^ and changes in dermal structure (decreased dermal echogenicity^12^ and increased thickness^13^). Therefore, an impaired skin barrier function renders the skin vulnerable and less resistant to mechanical stress, thus contributing to radiodermatitis exacerbation. As sun exposure leads to a reduction in skin barrier function, patients with head and neck cancer may exhibit a reduction in skin barrier function before radiotherapy initiation. However, their skin barrier function before radiotherapy initiation and its association with the sever radiodermatitis remains unclear.

Several studies have focused on severe radiodermatitis prevention in patients with head and neck cancer, but standard preventive care does not exist. The recommended preventive care includes moisturizing the irradiated area and avoiding mechanical stress during radiotherapy. A study in which skin moisturizing cream was applied from the first day of radiotherapy reported a 15.4% incidence of severe radiodermatitis but no significant difference relative to the control group.^14^ A systematic review of the preventive measures using topical non-steroidal agents showed no impact on the incidence of radiodermatitis, including upon trolamine application in two studies on patients with head and neck cancer.^15^ Similarly, a systematic review on the preventive use of Mepitel® Film on the skin to avoid mechanical stress demonstrated a reduction in moist desquamation, but the incidence rates reported were 20.5% and 45.5%.^16^ Therefore, the current recommended preventive care has failed to fully prevent the occurrence of severe radiodermatitis in patients with head and neck cancer, and optimal preventive care strategies should be developed.

To consider preventive care for severe radiodermatitis in patients with head and neck cancer, the cause of dermatitis at the irradiated site needs to be investigated and interpreted. For instance, in a study on preventive care for dermatitis surrounding malignant wounds in patients with breast cancer, there was a “radial shape matching the dressing,” caused by the presence of exudate.^17^ Therefore, frequent dressing changes or the use of a more absorbent dressing in cases of excessive exudate are recommended. Thus, clarifying the relationship between the morphological characteristics of severe radiodermatitis development and healing and factors that influence its severity will lead to the consideration of preventive care based on these factors.

The purpose of this study is to determine the morphological characteristics of the development and healing process of severe radiodermatitis in patients with head and neck cancer and to identify the skin barrier function associated with the development of the severe radiodermatitis.

## Methods

This prospective observational study comprised an exhaustive survey of patients undergoing radiotherapy for head and neck cancer at Hyogo Medical University hospital in Japan from October 2022 to March 2023. All investigations were conducted by a researcher certified in wound ostomy continence nursing.

### Participants

We included all patients diagnosed with head and neck cancer who received radiotherapy or chemoradiotherapy during the study period. The inclusion criteria were patients aged ≥ 20 years whose lymph node regions on both sides of the neck were included in the irradiation field. The non-inclusion criteria were a history of receipt of radiotherapy to the head and neck in the past, skin diseases, connective tissue disorders, and human immunodeficiency virus (HIV)-related diseases. The reason for non-including patients with HIV was that skin rashes may occur in the early stages of HIV. Eligible patients were selected by nurses at the research facility, and recruitment was conducted by the researcher. All participants were instructed by the facility nurse on skin care of the irradiated area (importance of gentle cleansing, moisturizing twice a day, and avoiding mechanical irritation) before the start of radiotherapy. A moisturizer (heparinoid lotion 0.3%) was prescribed by the radiation oncologist at the start of radiotherapy. During the radiotherapy period, the nurse checked the skin condition of the irradiated area once a week in case of radiodermatitis, and topical corticosteroid (hydrocortisone butyrate 0.1%) or non-steroidal agents (dimethyl isopropylazulene 0.033%) was prescribed by the radiation oncologist.

### Morphological characteristics

Investigations were conducted from before radiotherapy until 2 weeks after the end of the treatment or until radiodermatitis healed (epithelialization or pigmentation), and images of the irradiated area were obtained once a week after the start of radiotherapy. Images were acquired with a digital camera (IXY200, Canon, Tokyo, Japan) from approximately 30 cm away from the shooting site, capturing images from four directions: anterior, posterior, left, and right, including the mandible above the clavicle.

### Risk factors

The risk factors included patient-related and radiotherapy factors, which were collected from medical records. The patient-related factors were age, sex, BMI, serum albumin level, hemoglobin level, cancer type, disease stage, and smoking history. The radiotherapy factors were total dose, fractional dose, type of radiosensitizers, and use of topical corticosteroids. The skin surface dose was confirmed with the radiation oncologist based on the radiotherapy plan.

### Skin barrier function

Skin barrier function was assessed in terms of epidermal function and dermal structure before the start of radiotherapy. All assessments were conducted within a 5 ​× ​5-cm area between the right earlobe and the height of the thyroid cartilage to avoid overlapping assessment areas. Assessments were conducted at 22–25°C and 20% to 40% humidity.

Epidermal functions were assessed for stratum corneum hydration, TEWL, skin pH, sebum level, and skin microbiota. Stratum corneum hydration was measured using the Corneometer CM825 (Courage+Khazaka, Köln, Germany), TEWL was assessed using the Tewameter TM Hex (Courage+Khazaka), skin pH was assessed using the Skin-pH-Meter PH905 (Courage+Khazaka), and sebum level was determined using the Sebumeter SM815 (Courage+Khazaka). Skin microbiota was swabbed using the Levin method, and bacterial DNA was extracted from the samples using the ZymoBiomics DNA Miniprep Kit (Zymo Research Corp., California, USA) according to the manufacturer's instructions. Analysis was performed using the What's In My Pot program of the cloud analysis software EPI2ME (Oxford Nanopore Technologies Plc., Oxford, UK) on DNA sequences obtained by next-generation sequencing. We focused on the skin microbiota affecting radiodermatitis and other dermatitis and identified species abundance of the *Staphylococcus* genus. Following previous studies, the relative abundances of *Staphylococcus aureus* (*S. aureus*),^18^ *Staphylococcus epidermidis* (*S. epidermidis*),^19^ *Staphylococcus capitis* (*S. capitis*),^20^ *Staphylococcus caprae* (*S. caprae*),^20^ and *Staphylococcus hominis* (*S. hominis*)^19^ are displayed, and the abundances of other *Staphylococcus species* are designated as “*others*.”

Dermal structure was assessed for Subepidermal Low Echogenic Band (SLEB), and collagen density was evaluated using high-frequency ultrasonography (DermaScan, Cortex Technology, Aalborg, Denmark). Echogenicity was analyzed using the DermaScan analysis software (C USB Adv. Installation software rev.1.10.0.0.cs, Cortex Technology, Aalborg, Denmark) and expressed as “total intensity in %” values (relative to the average intensity of the specified range). SLEB was evaluated by a radiation oncologist and a dermatologist based on echo images according to the SLEB visual scoring (grade 1, subepidermal echolucent spots; grade 2, subepidermal echolucent patches; grade 3, continuous SLEB).^21^

### Data analysis

To examine the erosion phenomenon at irradiated sites in patients with head and neck cancer and the factors that influence it during the development and healing process, a qualitative analysis of the morphological characteristics of severe radiodermatitis was conducted using the qualitative sketch method. This is a useful approach for evaluating wound/skin conditions, their time course, and other related factors.^17^^,^^22^ Radiodermatitis was classified based on its morphological characteristics as severe radiodermatitis when there was “sloughing of the epidermis and exposure of the dermal layer, blister or vesicle formation, serous drainage”;^23^ this was evaluated based on erosion occurrence. Radiodermatitis was evaluated by a radiologist and a dermatologist, with the participants blinded. During the analysis, wound-care researchers provided supervision, and the validity of the results was confirmed by a radiation oncologist, a dermatologist, and a nurse specializing in radiotherapy.

For patients with severe radiodermatitis (severe group) and those without (non-severe group), the Mann–Whitney *U* test was used for continuous variables, and the Fisher's exact probability test was used for nominal variables regarding risk factors and skin barrier functions. Nonparametric methods were chosen for this study as normality could not be confirmed. The power (post-test) was confirmed to be 0.99, indicating that the number of participants was sufficient. Statistical analysis was performed using IBM SPSS Statistics ver.27 (IBM, Armonk, NY, USA), with a significance level set to *P* ​= ​0.05.

### Ethical considerations

This study was performed in line with the principles of the Declaration of Helsinki and its later amendments. Approval was granted by the Ethics Committee of Kanazawa University (IRB No. 711039), Hyogo Medical University (IRB No. 202205-176), Ishikawa Prefectural Nursing University (IRB No. 2023-339). All participants provided written informed consent.

## Results

### Participants

**Fig. 1:**
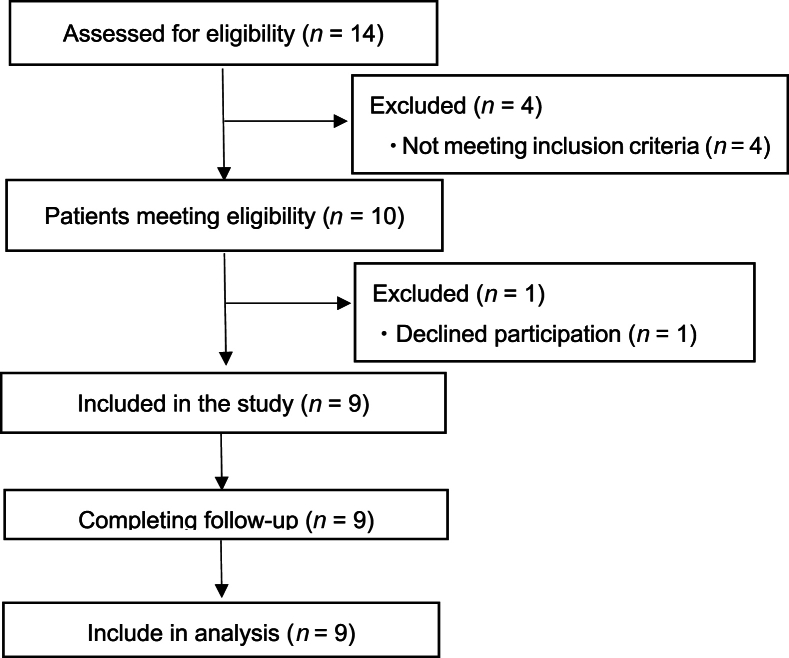
Study flowchart.

A total of nine patients participated in the complete study (Fig. 1). The total number of surveys was 88, with a median number of 9 (range 8–13) per person and a median follow-up of 61 (range 55–87) days. The participants had a median age of 57 (range 52–82) years, and 7 (77.8%) of them were males (Table 1). All patients received concurrent chemotherapy (cisplatin or cetuximab) and radiotherapy using VMAT with 6MV X-ray. Radiotherapy was administered once daily, with five fractions per week without discontinuation. The prescribed dose of radiotherapy was 70 Gy for gross tumor volume, 59.4 Gy for high-risk clinical target volume (CTV), and 54 Gy for low-risk CTV in 33 fractions, respectively. One patient received paclitaxel-carboplatin-cetuximab therapy before radiotherapy initiation. During the study period, all participants used moisturizers, and seven used topical corticosteroids.

**Table 1:** Risk factors associated with severe radiodermatitis.

	Factors	All patients (*n* ​= ​9)	Severe group (*n* ​= ​3)	Non-severe group (*n* ​= ​6)	*P* value
Patient-related factors	Age (years)	57 (52–82)	70 (57–82)	55.5 (52–77)	0.167
	Sex				
	Male	7 (77.8%)	3 (100.0%)	4 (66.7%)	0.500
	Female	2 (22.2%)	0 (0.0%)	2 (33.3%)	
	BMI (kg/m^2^)	21.09 (16.22–29.43)	21.09 (20.40–28.67)	21.43 (16.22–24.43)	0.714
	Cancer type				
	Larynx	2 (22.2%)	1 (33.3%)	1 (16.7%)	0.810
	Oropharynx	2 (22.2%)	0 (0.0%)	2 (33.3%)	
	Hypopharynx	4 (44.4%)	2 (66.7%)	2 (33.3%)	
	Tongue	1 (11.1%)	0 (0.0%)	1 (16.7%)	
	Disease stage				
	I	1 (11.1%)	0 (0.0%)	1 (16.7%)	0.290
	II	2 (22.2%)	1 (33.3%)	1 (16.7%)	
	III	3 (33.3%)	0 (0.0%)	3 (50.0%)	
	IV	3 (33.3%)	2 (66.7%)	1 (16.7%)	
	Smoker				
	Previous	8 (88.9%)	3 (100.0%)	5 (83.3%)	1.000
	Never	1 (11.1%)	0 (0.0%)	1 (16.7%)	
	Alb (g/L)	38 (30–43)	35 (30–40)	40 (37–43)	0.167
	Hb (g/L)	119 (95–157)	117 (95–137)	132 (105–157)	0.262
Radiotherapy factors	Total dose				
	69.96 Gy	8 (88.9%)	3 (100.0%)	5 (83.3%)	1.000
	60.00 Gy	1 (11.1%)	0 (0.0%)	1 (16.7%)	
	Fractional dose				
	2.12 Gy	8 (88.9%)	3 (100.0%)	5 (83.3%)	1.000
	2.00 Gy	1 (11.1%)	0 (0.0%)	1 (16.7%)	
	Radiosensitizer				
	Cisplatin	8 (88.9%)	2 (66.7%)	6 (100.0%)	0.333
	Cetuximab	1 (11.1%)	1 (33.3%)	0 (0.0%)	
	Topical corticosteroids				
	Use	7 (77.8%)	3 (100.0%)	4 (66.7%)	0.500
	Non-use	2 (22.2%)	0 (0.0%)	2 (33.3%)	

Continuous variables were compared using the Mann–Whitney *U* test and are presented as the median (range). Nominal variables were compared using the Fisher's exact test and are presented as *n* (%). BMI, body mass index; Alb, albumin; Hb, hemoglobin.

### Morphological characteristics and classification

**Fig. 2:**
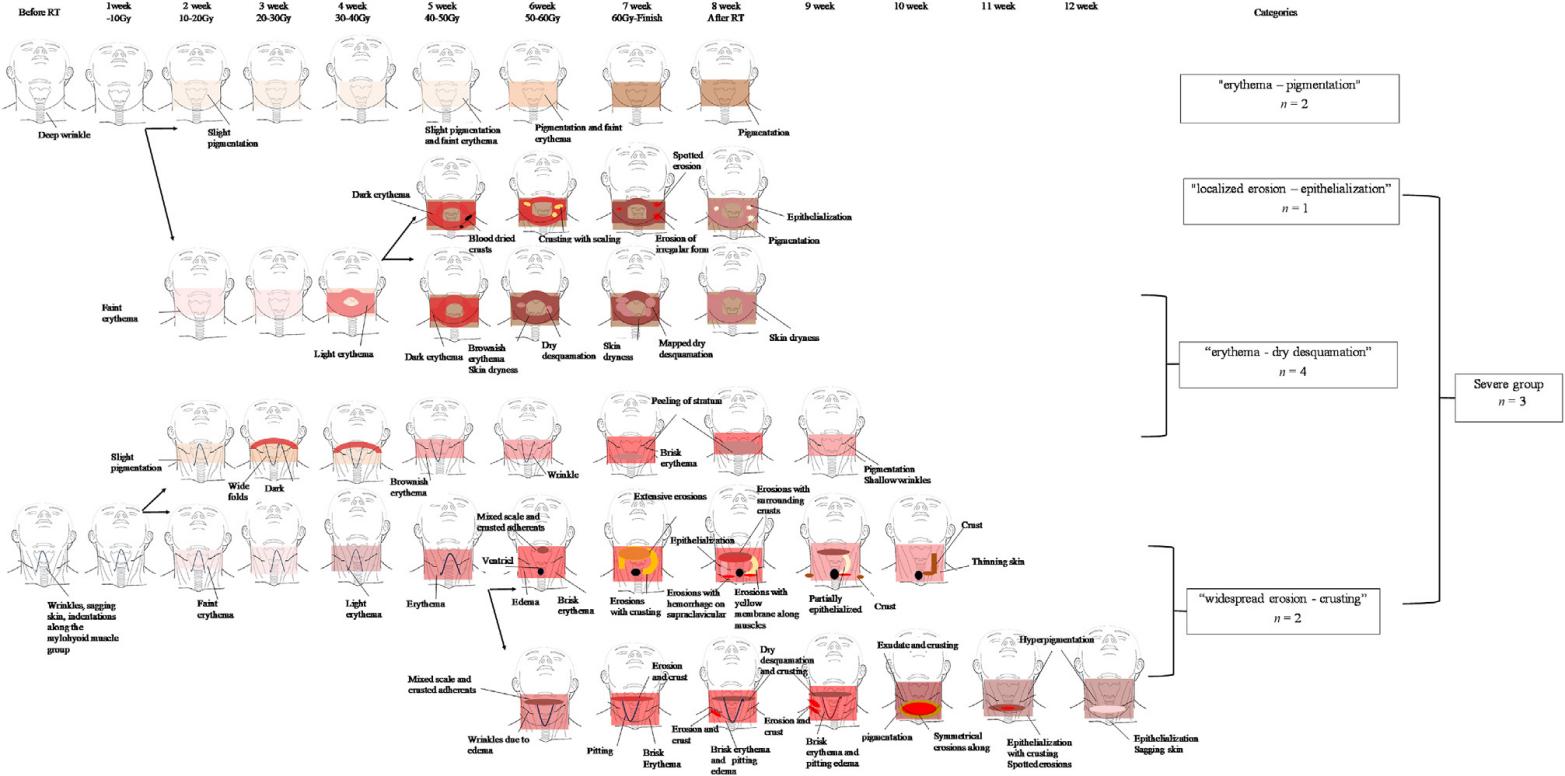
Pattern diagram and classification of morphological characteristics of radiodermatitis development and healing processes. RT, Radiotherapy.

The morphological characteristics of radiodermatitis were categorized into four: “erythema–pigmentation” (*n* ​= ​2), “erythema–dry desquamation” (*n* ​= ​4), “localized erosion–epithelialization” (*n* ​= ​1), and “widespread erosion–crusting” (*n* ​= ​2). “Localized erosion–epithelialization” and “widespread erosion–crusting” were classified as severe radiodermatitis, and a total of three patients had severe radiodermatitis. Fig. 2 shows a pattern diagram of the process for each category.

#### Morphological characteristics of “localized erosion – epithelialization”

This type was characterized by deep wrinkles on the anterior neck before radiotherapy initiation, but no sagging skin or fine wrinkles. At the end of radiotherapy, irregular erosions appeared in the neck wrinkles and collar height in the high-dose region. However, the erosions did not expand and healed by epithelialization after 1 week.

#### Morphological characteristics of “widespread erosion – crusting”

This type was characterized by fine wrinkles and sagging skin on the neck, indentations along the mylohyoid muscle group, and dry skin before radiotherapy initiation. After radiotherapy completion, extensive erosions with crusting due to exudate suddenly appeared in the highly inflamed, high-dose area. The cause was mechanical irritation from hospital gowns, wrinkles, and skincare. The erythema disappeared at the same time as widespread erosions developed, and epithelialization was observed progressively within a week, accompanied by crusting and scaling.

### Relationship between risk factors and severe radiodermatitis

A comparison of patient-related and radiotherapy factors between the severe and non-severe group showed no significant differences in any of the factors between the groups (Table 1).

### Relationship between severe radiodermatitis and skin barrier function

**Fig. 3:**
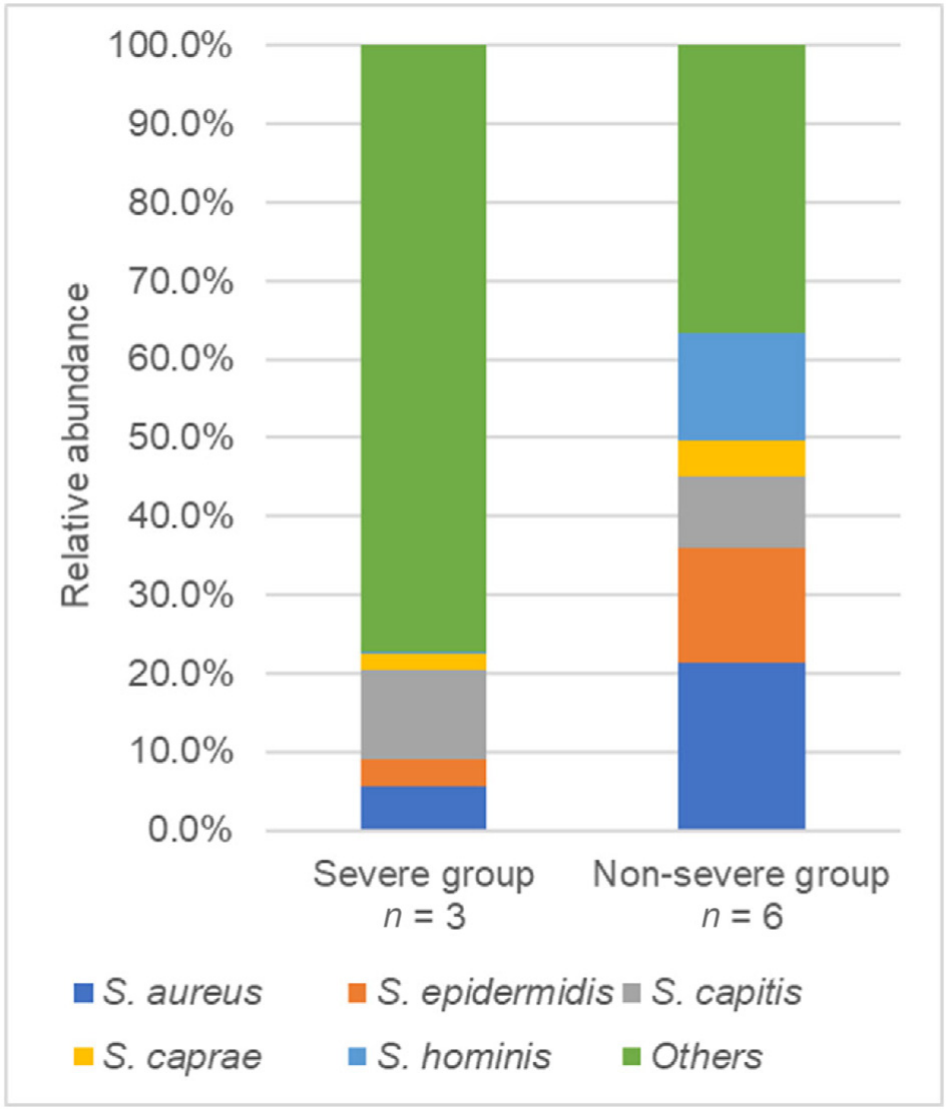
Comparison of skin microbiota in severe and non-severe groups. Microbial composition was shown using the relative abundance of bacteria in genus *Staphylococcus*, classified at the species level. *S. aureus*, *Staphylococcus aureus*; *S. epidermidis*, *Staphylococcus epidermidis*; *S. capitis*, *Staphylococcus capitis*; *S. caprae*, *Staphylococcus caprae*; *S. hominis*, *Staphylococcus hominis*.

**Fig. 4:**
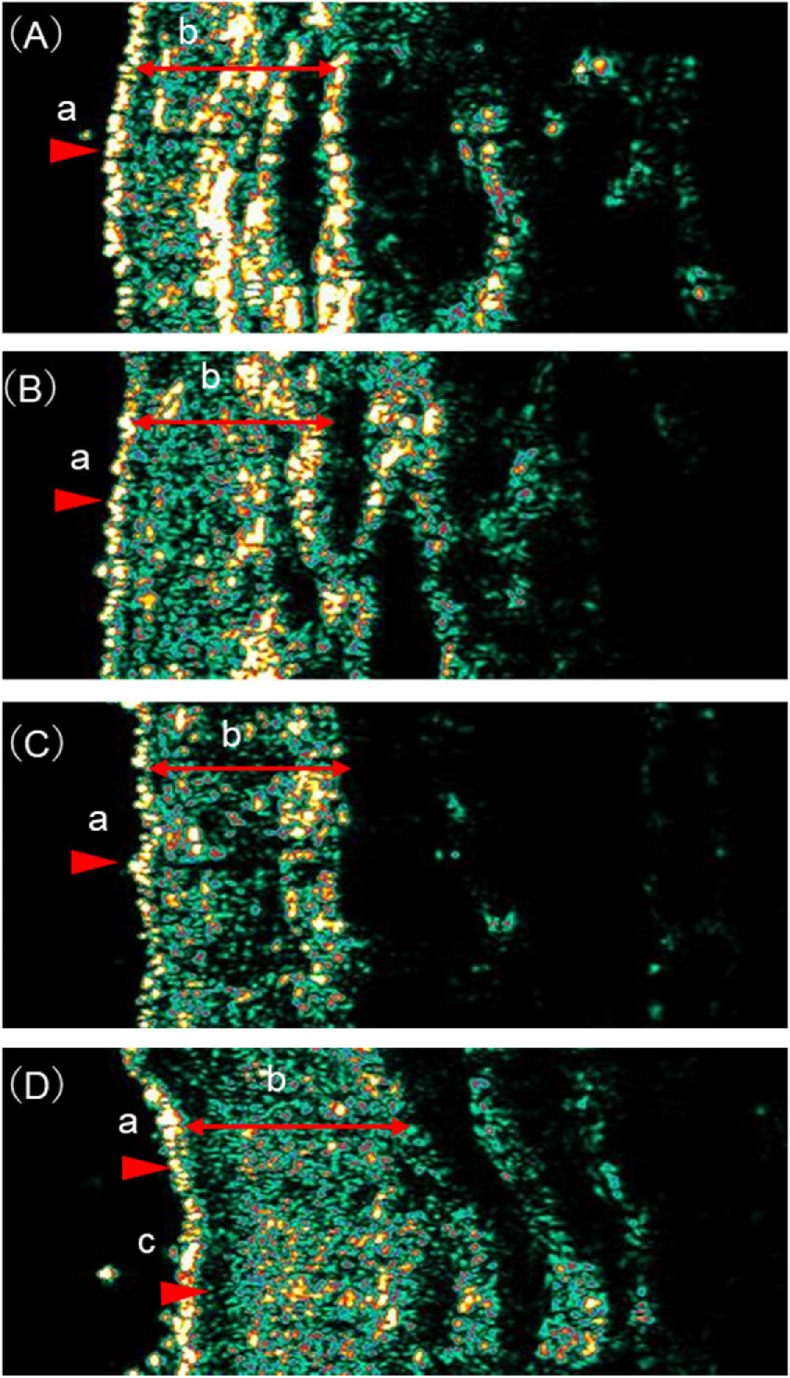
Representative ultrasonic images. One representative case from each group is provided: “erythema – pigmentation” in the non-severe group (A), “erythema–keratin peeling” in the non-severe group (B), “localized erosion–epithelialization” in the severe group (C), and “widespread erosion–crusting” in the severe group (D). The color scale of echogenicity is white-yellow-red-green-blue-black. a, epidermis, b, dermis, c, zonal hypoechoic areas in papillary dermis (SLEB grade 3). (C) and (D) Lower dermis echogenicity than (A) and (B) (*P* ​= ​0.036). Only (D) has a SLEB grade of 3. SLEB, subepidermal low echogenic band.

One participant who refused to be examined except for skin microbiota was included in the analysis only for skin microbiota. The relative abundance of *S. aureus* and *S. hominis* before the commencement of radiotherapy was significantly lower in the severe than in the non-severe group [*S. aureus*: severe-group median 4.8%, non-severe-group median 22.6% (*P* ​= ​0.024); *S. hominis*: severe-group median 0.2%, non-severe-group median 1.7% (*P* ​= ​0.024)] (Table 2 and Fig. 3). The severe group had also lower dermal echogenicity than the non-severe group [severe-group median 18.38%, non-severe-group median 24.94% (*P* ​= ​0.036)] (Table 2). Fig. 4 shows an example of ultrasonic images.

**Table 2:** Comparison of skin barrier functions of the severe and non-severe radiodermatitis group.

	Parameter	Severe group (*n* ​= ​3)	Non-severe group (*n* ​= ​6)	*P* value
Epidermis functions	Water content (a.u.)	54.3 (37.3–74.0)	44.3 (36.7–66.3)	0.571
	Sebum content (μg)	8 (4–40)	3 (1–7)	0.143
	Skin pH	4.79 (4.58–5.61)	6.18 (4.37–7.39)	0.393
	TEWL (g/h/m^2^)	11.77 (2.79–33.04)	9.65 (0.51–54.84)	1.000
	Skin microbiota *Staphylococcus* species level			
	*S. aureus*	0.048 (0.016–0.105)	0.226 (0.137–0.279)	0.024
	*S. epidermidis*	0.043 (0.013–0.048)	0.147 (0.015–0.326)	0.095
	*S. capitis*	0.066 (0.007–0.266)	0.092 (0.000–0.247)	1.000
	*S. caprae*	0.017 (0.006–0.041)	0.041 (0.009–0.094)	0.262
	*S. hominis*	0.002 (0.001–0.002)	0.017 (0.003–0.541)	0.024
	*Others*	0.820 (0.543–0.957)	0.368 (0.216–0.558)	0.048
Dermis structure	Dermal echogenicity (%)	18.38 (18.12–19.36)	24.94 (23.64–35.06)	0.036
	SLEB			
	Grade 1	1 (33.3%)	3 (60.0%)	0.286
	Grade 2	0 (0.0%)	2 (40.0%)	
	Grade 3	2 (66.7%)	0 (0.0%)	

Continuous variables were compared using the Mann–Whitney *U* test and are presented as the median (range). Nominal variables were compared using the Fisher's exact test and are presented as *n* (%). a.u., arbitrary units; TEWL, trans-epidermal water loss; SLEB, subepidermal low echogenic band; *S. aureus*, *Staphylococcus aureus*; *S. epidermidis*, *Staphylococcus epidermidis*; *S. capitis*, *Staphylococcus capitis*; *S. caprae*, *Staphylococcus caprae*; *S. hominis*, *Staphylococcus hominis*.

### Risk factors and skin barrier function characteristics of “widespread erosion–crusting”

Patients were > 70 years old, diagnosed with stage IV cancer, and had extensive areas of high skin surface dose. Analysis of morphological characteristics revealed fine wrinkles and sagging skin on the neck, skin dryness, indentations along the mylohyoid muscle group before radiotherapy initiation (Fig. 2), and grade 3 SLEB (contiguous SLEB) on echo imaging (Fig. 4).

## Discussion

To the best of our knowledge, this is the first study to show that the development of severe radiodermatitis in patients with head and neck cancer may be related to skin barrier function before radiotherapy initiation. The results suggest that skin care implemented after initiating radiotherapy is insufficient to prevent the occurrence of severe radiodermatitis and that care should be provided before therapy initiation.

### Association between severe radiodermatitis and skin barrier function

First, deterioration of the dermal structure at the irradiated site is possibly associated with severe radiodermatitis.

The severe group had lower echogenicity of the dermis than the non-severe group. The correlation between echogenicity and collagen fiber density in the dermis suggests that the decrease in echogenicity is caused by a structural change in the dermis due to decreased collagen fiber density.^24^ In addition, erosions occurred in areas subjected to mechanical stimuli such as collars of hospital gowns, skin wrinkles, and skin care. The dermal layer is composed of 70% collagen fibers and maintains the mechanical strength of the skin. Therefore, the erosions are likely to have occurred in patients with skin that was vulnerable to external forces owing to reduced collagen-fiber density before radiotherapy initiation, combined with skin fragilization due to the radiation factor and mechanical stimulation.

Second, changes in the composition of skin microbiota can be associated with severe radiodermatitis. In radiotherapy for breast cancer, severe radiodermatitis occurs in patients with a combined relative abundance of *S. epidermidis*, *S. hominis*, and *Cutibacterium acnes* of ≥ 5% before radiotherapy initiation.^19^ It is difficult to compare these results with those of the present study because they represent a percentage of the total skin microbiota, whereas the breast and neck skin microbiota composition is different. However, here, the relative abundance of *S. hominis* before the start of radiotherapy in the severe group was significantly lower (0.2%, *P* ​= ​0.024), and *S. epidermidis* was lower than in the non-severe group (4.3%), albeit not significantly. Furthermore, the relative abundance of *S. aureus* was also significantly lower in the severe group (4.8%, *P* ​= ​0.024) in this study. Therefore, impaired skin barrier function due to changes in the composition of the skin microbiota, such as decreased relative abundance of *S. hominis*, *S. epidermidis*, and *S. aureus* prior to the start of radiotherapy, can be associated with the development of severe radiodermatitis in patients with head and neck cancer.

### Association of extensive erosions with skin barrier function

Patients with extensive erosions had contiguous SLEB before radiotherapy. SLEB is observed in the skin as a result of aging and sun exposure, indicating degeneration of collagen and elastin in the papillary dermal layer and the presence of edema.^21^ Older patients with skin tear reportedly have a large SLEB area.^25^ In our study, patients with the “widespread erosion–crusting” type were > 70 years old. In addition, the neck skin is susceptible to sun exposure, which probably caused a decrease in the collagen density of the papillary dermis layer. Since the papillary dermal layer is responsible for connecting the epidermis and the dermis, the presence of SLEB could lead to epidermal desquamation and erosion expansion. Decreased tissue density of the dermal reticular formation and degradation of the collagen fiber structure of the dermal papillary layer lead to wrinkle formation.^26^ Therefore, sagging skin, fine wrinkles, and indentations along the mylohyoid muscle group at the irradiated area before the start of radiotherapy observed in this study represent signs of deterioration of the dermal structure. In the past, increased dermal thickness^12^^,^^13^ and decreased echogenicity of the deep dermis^13^ have been reported after radiotherapy for breast cancer, and radiotherapy-induced changes in the structure of the dermis led to skin fragility. However, our findings suggest that patients with neck sagging, fine wrinkles, and indentations along the striated muscle groups before radiotherapy initiation have deteriorated dermal structure, which led to extensive erosions upon radiotherapy.

### Implications for nursing practice and research

Our study established that observing the neck skin before radiotherapy initiation can predict severe radiodermatitis. Patients with sagging skin, fine wrinkles, and indentations along the mylohyoid muscle group on the neck before the commencement of radiotherapy may develop extensive erosions. This leads to the identification of high-risk patients with severe radiodermatitis. For patients with these morphologic characteristics, the previously recommended care of moisturization and mechanical irritation avoidance during radiotherapy to preserve the skin barrier function even before radiotherapy initiation is essential. Specifically, we recommend applying moisturizers to improve the skin microbiota condition^27^ and ingesting collagen peptides to improve the dermal structure.^24^ These suggestions may help prevent severe radiodermatitis in patients with head and neck cancer.

### Limitations

This study has some limitations. First, the study was conducted at a single institution with a small number of participants, possibly influencing the results regarding the severity and morphological characteristics of radiodermatitis. However, it is valid in that the results were checked with radiation therapy experts for generalizability. The results were also used after confirming the post-hoc power using the effect size of dermal echogenicity. Next, the lack of sufficient data did not allow the results to be discussed extensively. One patient had a sagging neck, fine wrinkles, and indentations along the mylohyoid muscle group before radiotherapy initiation but did not present severe radiodermatitis. This patient refused to have their skin barrier function investigated other than their skin microbiota; therefore, the cause of the lack of erosion enlargement was unknown. Finaly, due to the limited number of participants, sub-analysis could not be performed accounting for confounding factors such as age and topical corticosteroids. In the future, it will be necessary to increase the number of participants and address confounding factors through statistical analysis, as well as include a control group to strengthen the findings.

## Conclusions

This study identified the morphological characteristics of severe radiodermatitis to be “localized erosions–epithelialization” and “widespread erosions–crusting”. Patients with head and neck cancer and severe radiodermatitis who develop extensive erosions were found to have impaired skin barrier function even before the commencement of radiotherapy. Preventive care for severe radiodermatitis should not only include conventional moisturizing and avoidance of mechanical stimulation during radiotherapy. It is suggested that the skin barrier function, including the state of the skin microbiota and dermal structure, be improved prior to the start of radiotherapy.

## CRediT authorship contribution statement

**Nao Miyamae**: Conceptualization, Methodology, Collected the data, Formal analysis, Data Curation, Writing – Original draft, Writing – Review & Editing, Funding acquisition. **Kazuhiro Ogai**: Investigation, Resources, Writing – Review & Editing. **Mao Kunimitsu**: Formal analysis, Writing – Review & Editing. **Masayuki Fujiwara**: Validation, Writing – Review & Editing. **Makoto Nagai**: Validation, Writing – Review & Editing. **Shigefumi Okamoto**: Writing – Review & Editing, Supervision. **Mayumi Okuwa**: Writing – Review & Editing, Supervision. **Makoto Oe**: Conceptualization, Methodology, Formal analysis, Writing – Review & Editing, Supervision, Project administration. All authors had full access to all the data in the study, and the corresponding author had final responsibility for the decision to submit for publication. The corresponding authors attest that all listed authors meet authorship criteria and that no others meeting the criteria have been omitted.

## Ethics statement

This study was performed in line with the principles of the Declaration of Helsinki and its later amendments. Approval was granted by the Ethics Committee of Kanazawa University (IRB No. 711039), Hyogo Medical University (IRB No. 202205-176), Ishikawa Prefectural Nursing University (IRB No. 2023-339). All participants provided written informed consent.

## Data availability **statement**

The data that support the findings of this study are available from the corresponding author, MO, upon reasonable request.

## Declaration of generative AI and AI-assisted technologies in the writing process

No AI tools/services were used during the preparation of this work.

## Funding

This work was supported by JSPS KAKENHI (Grant No. JP23K09996). The funder had no role in considering the study design or in the collection, analysis, interpretation of data, writing of the report, or decision to submit the article for publication.

## Declaration of competing interest

The authors declare no conflict of interest.
